# Unravelling the myriad physiologic roles of transthyretin: critical considerations for treating transthyretin amyloidosis

**DOI:** 10.1080/07853890.2025.2536755

**Published:** 2025-07-27

**Authors:** Morie A. Gertz, Mandar A. Aras, Nicole Bart, Thomas H. Brannagan III, Jan M. Griffin, Amy R. Kontorovich, Andrew M. Rosen

**Affiliations:** ^a^ Division of Hematology, Mayo Clinic, Rochester, MN, USA; ^b^ Department of Medicine, Division of Cardiology, UCSF Medical Center, University of California San Francisco, San Francisco, CA, USA; ^c^ Department of Cardiology, St Vincent’s Hospital, Sydney, NSW, Australia; ^d^ School of Clinical Medicine, Faculty of Health and Medicine, The University of New South Wales, Sydney, NSW, Australia; ^e^ The Victor Chang Cardiac Research Institute, Sydney, NSW, Australia; ^f^ Department of Cardiology, Brigham and Women’s Hospital, Boston, MA, USA; ^g^ Harvard Medical School, Boston, MA, USA; ^h^ Department of Neurology, Columbia University, Vagelos College of Physicians and Surgeons, New York, NY, USA; ^i^ Department of Medicine, Division of Cardiology, Medical University of South Carolina, Charleston, SC, USA; ^j^ Fuster Heart Hospital and Cardiovascular Research Institute, Icahn School of Medicine at Mount Sinai, New York, NY, USA; ^k^ BridgeBio Pharma, Inc, San Francisco, CA, USA

**Keywords:** Transthyretin, TTR, ATTR, amyloidosis, Alzheimer’s disease, neurodegenerative disorders, cardiovascular disease, cerebrovascular disease, genetics, prealbumin

## Abstract

**Background:**

Transthyretin (TTR) is a highly conserved protein with crucial and broadly protective physiologic roles across organ systems and diseases. Evidence shows that TTR contributes to neuroprotection, cognition, glucose regulation, pregnancy, muscle development, and bone mineralization. In several disease states, including diabetes, Alzheimer’s disease, Lewy body dementia, cerebrovascular disease, and osteoporosis, higher TTR levels may be protective. Numerous studies have shown that low levels of TTR are associated with increased mortality overall and in relation to cardiovascular disease and several malignancies.

**Purpose:**

There is a growing portfolio of approved and investigational transthyretin amyloidosis (ATTR) treatments that differ in their mechanisms and effects on circulating TTR. When selecting an ATTR therapy, clinicians must decide whether to stabilize and preserve TTR and its functions or knockdown and drastically reduce TTR. This review summarizes the vital physiologic roles of TTR in health and disease. We consider the potential effects on normal biologic pathways that may occur while therapeutically suppressing TTR and discuss clinical decisions concerning ATTR therapies in the context of the summarized literature.

**Discussion:**

TTR is essential for a broad range of physiologic processes and may confer clinically protective effects in neurologic and other organ systems. While a link between low TTR and severe disease and mortality is well established, it remains unclear whether long-term TTR suppression *via* ATTR therapies increases risk of disease. Clinical decisions in ATTR, however, should reflect the current understanding of the roles of TTR and the patient’s clinical history.

**Conclusion:**

TTR serves vital physiologic roles across organ systems. Given its clinically protective properties, continued investigation into the potential long-term impact of TTR suppression *via* knockdown or gene editing therapies is prudent. ATTR treatment selection should reflect an awareness of the physiologic importance of TTR, as well as consideration of the potential long-term impact of chronic TTR suppression.

## Introduction

Transthyretin (TTR) is a tetrameric protein that serves critical physiologic functions across multiple organ systems while also harbouring the potential pathogenic precursor of transthyretin amyloidosis (ATTR) [1]. TTR is produced primarily by the liver and in smaller amounts by the choroid plexus and retinal epithelium; it subsequently circulates in serum, cerebrospinal fluid (CSF), and ocular tissues [1–5]. While TTR has garnered significant attention due to its apparent role in amyloid pathogenesis, there is also considerable evidence that it plays an active and often protective role across various organ systems [1,6–8]. TTR levels are typically lower in females and show a negative correlation with C-reactive protein levels [9]. In contrast, TTR levels increase alongside higher body mass index, systolic and diastolic blood pressure, and levels of total cholesterol, albumin, triglycerides, and creatinine [9]. Additionally, TTR levels tend to decrease as a person ages [10].

In ATTR, destabilization of the native TTR tetramer leads to its dissociation into monomers, which misfold and aggregate as amyloid fibrils, predominantly in the myocardium and peripheral nerves, causing cardiomyopathy and polyneuropathy, respectively [11–14]. ATTR amyloidosis, whether it impacts the heart or the nervous system, is a progressive, highly debilitating, and fatal disease [15–17]. With increased disease awareness, focused screening, availability of targeted therapies, and adoption of non-invasive diagnostic techniques, patients are being diagnosed with ATTR and initiating treatment earlier and, as such, are living longer and with improved quality of life [18–22]. Several therapies for the treatment of ATTR are approved or in development, including those that preserve the function of TTR by stabilizing its tetrameric form and those that reduce TTR levels using TTR messenger RNA (mRNA) knockdown or *TTR* gene editing (see ‘Treatment landscape’) [18,23,24]. The improving prognosis for ATTR [18,19] means patients will live longer, with more prolonged exposure to ATTR therapies. Given the evidence broadly supporting a protective role for TTR across several organ systems and diseases, physicians and patients must consider the potential long-term clinical consequences of stabilizing and preserving TTR or suppressing TTR levels in patients receiving treatment for ATTR amyloidosis. The evidence signals that it is imperative to research the potential long-term consequences of reducing or eliminating TTR with targeted therapies [1,25].

This narrative review aims to elucidate the myriad functions through which TTR actively influences multisystem health and disease [1,26]. We present a comprehensive summary of the literature to date and highlight important areas that warrant further research. Moreover, we discuss the evidence in the context of clinical decisions regarding ATTR treatment.

## Literature search and study selection

A targeted literature search was conducted in PubMed in June 2024, limited to publications within the last 10 years (June 2014 to June 2024), to identify literature related to the role of TTR in health and disease. Keywords included ‘transthyretin’ in various combinations with ‘disease,’ ‘health,’ ‘role,’ ‘neuroprotection,’ ‘protection,’ ‘function,’ and ‘pathophysiology.’ Articles were screened based on title, abstract, and/or full text to determine their relevance to the topic. Both original research publications and review articles were included. Additional targeted searches in PubMed and the authors’ own libraries were conducted to ensure a comprehensive review of the literature.

## Role of TTR in human physiology and disease

### Overview

TTR was first identified in the 1950s when scientists discovered a serum protein that binds thyroxine, initially naming it thyroxine-binding prealbumin [27]. The structure of TTR, which is tetrameric, and the allosteric mechanism behind its two thyroxine-binding sites were later revealed through crystallography [10,27,28]. In the early 1980s, thyroxine-binding prealbumin was renamed transthyretin because of its role in transporting vitamin A alongside retinol-binding protein [10,27]. Research into the molecular genetics of TTR began in the 1980s, and inherited variants associated with variant-driven ATTR, with either polyneuropathy or cardiomyopathy, were uncovered over the following years [21,27,29–31]. Today, both wild-type (ATTRwt) and variant (ATTRv) transthyretin amyloidosis are recognized and increasingly diagnosed [32]. Approved therapies for ATTR that either stabilize and preserve TTR or knockdown and diminish the protein are available; therefore, a greater awareness of the physiologic roles of TTR is of utmost importance [18]. TTR circulates as a tetramer consisting of four identical β-strand-rich monomeric subunits that are arranged to form a hydrophobic central pocket with two binding sites [1,8,33]. TTR binds thyroxine (T_4_) and retinol-binding protein, enabling it to transport T_4_ and retinol (vitamin A) systemically [1,34].

**Figure 1.**
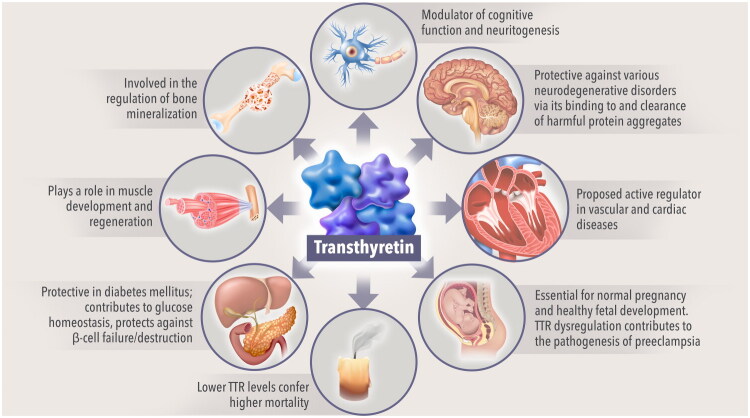
**The putative role of TTR in health and disease.** Evidence from animal models has shown an effect of TTR on maintaining ‘normal’ memory, with the absence of TTR impairing spatial learning and accelerating cognitive decline typically associated with aging [35–38]; TTR also promoted neuritogenic activity in the central and peripheral nervous systems in animal studies *in vitro* and *in vivo* [39–42]. TTR may have neuroprotective activities in Alzheimer’s disease, Parkinson’s disease, and TDP-43-associated neuropathies by binding, degrading, and/or clearing harmful protein aggregates [43–47]. TTR has been implicated in cardiovascular health in epidemiological studies [9,48,49]. Regulation of TTR expression appears to be essential for the maintenance of healthy pregnancy and normal fetal development in mice [50,51]. Lower levels of TTR have been linked to higher mortality in the general elderly population, as well as several oncological diseases, respiratory diseases, cardiovascular diseases, and ATTR [9,52–57]. In *ex vivo* pancreatic islet preparations, TTR had a role in glucose-induced insulin release and offered protection against β-cell apoptosis [58]. In *in vitro* animal models, TTR appeared to participate in the development and regeneration of muscle cells [59,60]. Destabilized forms of TTR distorted the morphology of calcium carbonate crystals *in vitro*, suggesting the involvement of TTR in healthy bone mineralization [61]. ATTR, transthyretin amyloidosis; TDP-43, trans-activation response DNA-binding protein 43 kDa; TTR, transthyretin.

Beyond this well-established role, TTR serves myriad vital physiologic functions across organ systems [1,26]. The interaction of TTR with numerous proteins and pathways underscores its delicate and crucial homoeostatic role in both health and disease (Figure 1) [1,26]. As described here, there is evidence that TTR plays a role in neuroprotection, cognitive function, glucose homeostasis, pregnancy, myogenesis, and bone mineralization [50,51,61–64]. The multisystemic impact of this highly conserved protein is also evidenced by its ability to reduce the risk of some pathologic processes or enhance protective processes in the context of several diseases [39,43,65].

The ability of TTR to carry out its physiologic functions depends on the stability of its tetrameric structure [7,21,30]. In ATTR for example, conformational alteration of TTR arises from kinetic or thermodynamic instability driven by either a heritable amyloidogenic variant of the *TTR* gene, of which more than 130 such variants have been discovered to date, age-related factors, or epigenetics [6,21,29,30,66–68]. The extent of TTR instability varies across *TTR* genotypes, as well as between the ATTRv and ATTRwt forms of the disease [69]. Greater TTR instability is associated with a more severe ATTR disease phenotype and a worse prognosis [69]. In contrast, the *TTR* gene variants p.T139M (previously referred to as T119M) and p.R124H (previously referred to as R104H) confer stabilization of the tetramer and, therefore, have a downstream protective effect against ATTR [70–74]. It is important to note that destabilization of the TTR tetramer decreases levels of circulating TTR, which evidence suggests incurs deleterious health outcomes [49].

The following sections of this comprehensive review aim to illuminate the diverse roles of TTR across physiologic functions and disease [1,26].

### Alzheimer’s disease and other neurodegenerative disorders

Alzheimer’s disease (AD), the most common and increasingly prevalent form of dementia [75,76], is characterized biochemically by the deposition of extracellular, neurotoxic aggregates of amyloid beta (Aβ) that polymerize to form fibrillar plaques in the brain [77,78]. The 5-year mortality of patients with AD is estimated at 35%, and comorbidities include hypertension, diabetes, and stroke [79]. In AD, amyloid precursor protein is hydrolysed by β-secretase, resulting in the production of Aβ monomers, especially the Aβ42 isoform, which has a greater propensity to aggregate than the Aβ40 isoform [80,81]. Oligomerization of these monomers produces a highly neurotoxic form of Aβ [82,83]. Other pathologic features contributing to disease progression include neurofibrillary tangles of hyperphosphorylated tau protein, changes in the lymphatic system and the peripheral immune system, and diminished integrity of the blood–brain barrier (BBB), the latter of which has been shown to increase vascular permeability [78,84,85].

Several studies have shown that patients with AD have reduced levels of TTR in their CSF [86] and plasma [87], while others have shown that CSF levels can be elevated [88,89]. Some studies support that reduced TTR levels negatively correlate with disease progression [46,87,90,91]; patients with mild cognitive impairment, which often precedes AD, tend to have higher serum TTR levels, which reduce as the disease progresses [62]. However, with the variability of reported results, it is generally agreed that CSF TTR is not a reliable biomarker for the diagnosis of AD [89,92,93]. Nevertheless, it is accepted that TTR stability is decreased in patients with AD, and the tetrameric-to-monomeric ratio is lower than in age-matched controls [94].

**Figure 2.**
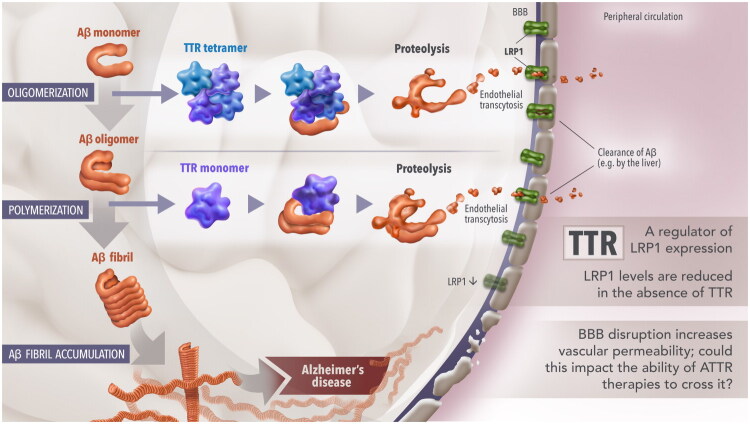
**Role of TTR in Alzheimer’s disease.** TTR has proteolytic activity capable of cleaving Aβ monomers and oligomers, potentially contributing to their clearance [46]. *In vitro* results from a mouse model of Alzheimer’s disease showed that TTR tetramers bind Aβ monomers, preventing Aβ oligomerization [46]. Results from laboratory assays indicated that TTR monomers bind Aβ oligomers, inhibiting their polymerization to fibrils [95]. Tetrameric TTR increased transmembrane LRP1 levels, potentially contributing to clearance of Aβ from the brain [43,94]. BBB dysfunction, which is characteristic of Alzheimer’s disease, results in increased in vascular permeability [84,85]; it would be interesting to determine whether this increased permeability extends to the ability of ATTR therapies to cross the BBB. Aβ, amyloid beta; ATTR, transthyretin amyloidosis; BBB, blood–brain barrier; LRP1, low-density lipoprotein receptor-related protein 1; TTR, transthyretin.

TTR is neuroprotective and may be considered a neurotrophic factor given its role in stimulating neurite outgrowth and promoting neurogenesis [39]. The neuroprotective role of TTR is perhaps most evident in AD, in which it has been shown to bind, sequester, transport, and proteolyze Aβ (Figure 2) [43,46,94,96–100]. *In vitro*, the binding of TTR to Aβ can both inhibit fibril formation and degrade existing fibrils *via* the proteolysis of Aβ into shorter nonamyloidogenic fragments [46,97,98]. The relative importance of monomeric versus tetrameric TTR has been investigated *in vitro* using wild-type TTR and genetically engineered TTR mutants to determine binding affinities to Aβ and effectiveness in inhibiting Aβ aggregation [95,101–103]. Overall, the evidence indicates that stable TTR tetramers bind Aβ monomers, preventing their oligomerization, and TTR monomers (or destabilized tetramers) bind Aβ oligomers, thereby inhibiting their polymerization to fibrils [95,104]. Increased stability of tetrameric TTR, either *via* a *TTR* variant or small molecule compounds, increases Aβ binding and proteolytic activity [105]. In humans, the manyfold higher concentrations of TTR tetramers than monomers suggests that the tetrameric form is likely to have the predominant protective role in AD [96,104].

In addition to direct inhibition of Aβ aggregation, tetrameric TTR contributes to the clearance of Aβ from the brain through the transmembrane low-density lipoprotein receptor-related protein 1 (LRP1) (Figure 2) [94]. LRP1 is the main efflux receptor for transcytosis of Aβ across the BBB and mediates Aβ clearance through endocytosis and subsequent lysosomal degradation by hepatocytes in the periphery and in neurons and astrocytes in the brain [94,96,106,107]. Research has shown that TTR facilitates the transport of Aβ from the brain and increases its uptake by the liver (the primary organ for clearing Aβ in the periphery [108]) using LRP1 [43]. *In vitro*, the stable tetrameric form of TTR facilitates cellular uptake of Aβ and increases LRP1 levels [94]. It has been shown that TTR can cross the BBB in mice, and an *in vitro* investigation has shown that TTR can transport Aβ in the brain-to-blood direction [5,43,94]. It is proposed that tetrameric TTR is involved in the transport and clearance of Aβ from the brain [43,94]. Evidence from mouse models of AD have demonstrated that treatment with iododiflunisal, a TTR stabilizer that crosses the BBB, decreases the rate of Aβ deposition in the brain and may improve cognitive function [109,110].

Vascular defects are an early feature in the progression of AD and precede Aβ plaque formation [111]. Proangiogenic properties of TTR may be another mechanism by which it can moderate neurodegenerative disease [112]. It has been shown that TTR increases the expression of key angiogenic molecules by endothelial cells, including interleukins (IL-) 6 and 8, angiopoietin 2, and vascular endothelial growth factor, *in vitro* [112]. In the brains of AD transgenic mice, microvessels are shorter and have thicker basement membranes when TTR expression is reduced [112]. Moreover, when such mice were treated with a stabilizer of tetrameric TTR, basement membranes were less thick and vessels were longer than in untreated control mice [112].

The clinically protective role of TTR may extend to other neurodegenerative disorders and core cognitive functions such as learning and memory [35,36,44,45,47]. Evidence supporting this broader function derives from the interaction of TTR with pathogenic proteins, such as α-synuclein, and TTR-mediated increases in neuritogenesis and angiogenesis [39,47,112]. TTR has been implicated in several neurodegenerative disorders, such as Parkinson’s disease, which is a Lewy body disorder [47]. Lewy bodies are insoluble protein inclusions that predominantly comprise α-synuclein, the oligomers of which are neurotoxic [113,114]. TTR has been shown to facilitate *in vitro* proteolysis of specific conformations of misfolded α-synuclein into aggregation-incompetent fragments, suggesting that TTR has a direct neuroprotective role in Lewy body diseases [47].

Other neurodegenerative disorders, such as amyotrophic lateral sclerosis and certain subtypes of frontotemporal lobar degeneration (FTLD), are associated with toxic aggregations of trans-activation response DNA-binding protein 43 kDa (TDP-43) [115]. TDP-43 proteinopathy results in the redistribution of nuclear TDP-43 to the cytoplasm, where it accumulates and is sequestered as cytoplasmic aggregates; nuclear aggregates of TDP-43 are also observed [116–119]. It has been reported that TTR coaggregates with TDP-43, as assessed in both the postmortem brains of patients with TDP-43–associated FTLD and an FTLD transgenic mouse model [44]. The authors found that TTR promoted the degradation and clearance of TDP-43 aggregates by: (1) escorting and facilitating the docking of TDP-43 aggregates to autophagosomes for degradation; and (2) promoting the upregulation of autophagy *via* activating transcription factor 4 [44,45]. This evidence suggests that TTR is protective in TDP-43–associated neurodegenerative disorders, such as FTLD.

In summary, the data suggest a multifaceted and clinically protective role of TTR in several neurodegenerative diseases. In AD for example, in which the evidence is most robust, TTR was shown to bind, sequester, transport, and degrade harmful Aβ fibrils, while promoting angiogenesis [5,43,94,96,106,112]. In addition, data suggest a diverse and protective role of TTR in Lewy body disorders and TDP-43–associated neuropathologies that involve proteolysis and the promotion of degradation and clearance of harmful protein aggregates [45,47].

### Cognitive function and neuritogenesis

Transthyretin has an active role in neuritogenesis and angiogenesis, which support neuroplasticity, recovery from injury (whether it be stroke or traumatic brain injury), cognitive function, and healthy autonomic, neuromuscular, and somatosensory function [120–125]. Studies have shown that changes in circulating TTR levels track the progression from mild cognitive impairment to dementia in the older population [62,87,90,91,126–128], though not all studies have replicated these findings [89,93].

Evidence from animal models has shown the direct effects of TTR on memory, spatial learning, and cognitive decline [37,38]. In mouse models, the well-established function of TTR as a carrier for retinol-binding protein appears to be critical for the involvement of TTR in maintaining ‘normal’ memory [35]. One study found lower levels of *TTR* expression in the hippocampus of aged, memory-impaired rats than in aged, memory-unimpaired rats, as well as memory deficits in TTR-knockout mice [35]. Another study in TTR-knockout mice showed impaired spatial learning and loss of cortical and hippocampal neurons, which were attributed to TTR but likely not related to its transport of retinol-binding protein [36]. Additionally, the absence of TTR appears to accelerate the decline in cognitive performance that is typically associated with aging in these mouse models [37,38].

Neuritogenesis, which refers to the development, extension, and branching of neurites, is a critical step in building a network of neurons that can communicate effectively [129]. TTR has neuritogenic properties in both the central nervous system [39] and peripheral nervous system [40–42] that are believed to be independent of its transport of retinol-binding protein or T_4_ [39,41]. The significance of this in the peripheral nervous system is uncertain, since knockdown of TTR by TTR silencers for 3–5 years results in the improvement or stabilization of peripheral neuropathy in patients with ATTR [130,131]. It has been hypothesized that neuritogenic activity in the brain might be one mechanism through which TTR has a beneficial impact on learning and memory [39]. Models of focal cerebral ischaemia have demonstrated TTR to have a direct neuroprotective role by promoting neurite outgrowth and survival in mouse hippocampal neurons subjected to ischaemic conditions [39]. In summary, TTR may act as a neuromodulator and aid important cognitive functions, such as learning and memory, while offering clinical protection from neurodegenerative disease [37–39,87,91].

### Cerebrovascular and cardiovascular disease

TTR likely serves as a key regulator of cerebrovascular and cardiovascular diseases [39,132]. Research has shown that low levels of TTR increase the risk for heart failure [48], as well as all-cause and cardiovascular death compared with normal TTR levels [9]. In addition, TTR levels have been shown to be lower in the serum and peripheral blood mononuclear cells isolated from patients with coronary artery disease compared with healthy controls [132]. Serum TTR levels are considered a marker of TTR tetramer stability, and in patients with ATTR cardiomyopathy (ATTR-CM), low serum TTR levels are associated with tetramer dissociation into monomers, which misfold and aggregate as amyloid fibrils in the myocardium [11,12,14,133]. Although the physiologic mechanism responsible for the protective cardiovascular effects of TTR requires further study, protein interaction analysis indicates a role in cholesterol transport, atherogenic lipoprotein binding, and lipid metabolism [132].

A study using data from two prospective cohort studies of the Danish general population supported the association between incident heart failure and low TTR levels, suggesting that TTR levels may act as a biomarker for heart failure [48]. Interestingly, when people with different *TTR* variants were compared, TTR levels decreased and heart failure risk increased in descending order of the tetramer stability of the variant; carriers of the known stabilizing *TTR* variant p.T139M had the lowest disease risk. In addition, the risks of cardiovascular mortality and all-cause mortality were increased in carriers of *TTR* destabilizing variants [49]. These data suggest an association between cardiac health and TTR conformation [48]. Observations in the UK Biobank showed that carriers of the *TTR* pathogenic variant p.V142I have reduced TTR levels compared with noncarriers, with low TTR levels associated with increased risks of heart failure, cardiovascular disease, atherosclerotic cardiovascular disease, all-cause mortality, and cardiovascular mortality [9].

Using data from the above Danish cohort studies, Hornstrup et al. demonstrated that heterozygotes for the p.T139M *TTR* variant had 17% higher TTR levels than noncarriers [54]. Carriers also exhibited 20% higher total T_4_ levels, attributed to the increased binding affinity of this more stabilized TTR protein for T_4_. This genetically mediated stabilization was associated with decreased risks of cerebrovascular disease and all-cause death, as well as a higher age at death in those who died owing to cerebrovascular disease. Of note, these observations were not reproduced in a more recent and larger population-based (UK Biobank) study focused on white individuals, in which p.T139M was not associated with protection against cerebrovascular disease or death, and the mean age at all-cause mortality did not differ between carriers and noncarriers [134].

### Metabolism and metabolic disease

Type 1 diabetes is believed to be caused by the autoimmune destruction of the insulin-producing islets of Langerhans β-cells [135]. In patients with type 1 diabetes, serum tetrameric TTR concentrations are lower and monomeric TTR concentrations are higher than in healthy controls [58]. In *ex vivo* pancreatic islet preparations, tetrameric TTR plays a role in the normal coupling of glucose stimulation with insulin secretion by pancreatic β-cells *via* its direct effects on glucose-induced electrical activity and voltage-gated calcium (Ca^2+^) channels [58]. Tetrameric TTR also partly protects against apolipoprotein CIII-induced β-cell apoptosis, whereas monomeric TTR does not impact insulin release or β-cell integrity [58]. Taken together, the conversion of tetrameric TTR to its monomeric form has been proposed to mediate the development of β-cell failure/destruction in type 1 diabetes [58].

**Figure 3.**
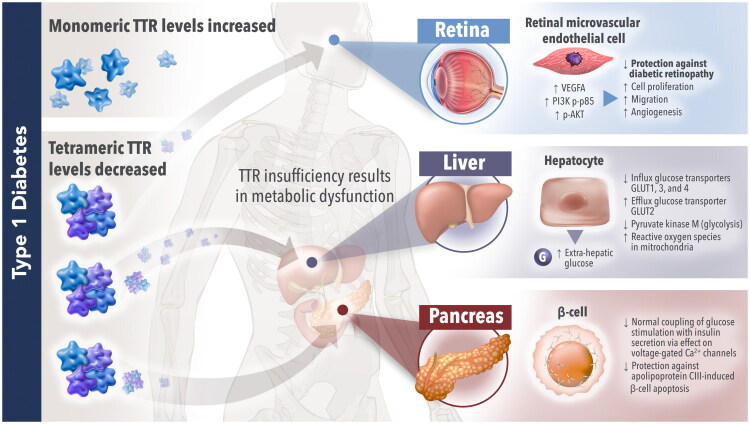
**Role of TTR in type 1 diabetes.** In cultures of human retinal microvascular endothelial cells, TTR mitigated the damaging cell proliferation, migration, and angiogenesis effects of high glucose concentration by inhibiting the VEGFA/PI3K/AKT pathway [136]. In mice, TTR insufficiency caused elevated plasma glucose concentration, decreased expression of influx glucose transporters GLUT1, GLUT3, and GLUT4, increased expression of efflux glucose transporter GLUT2, reduced levels of the glycolytic enzyme pyruvate kinase M, and impaired mitochondrial activity [63]. In *ex vivo* pancreatic islet preparations, the TTR tetramer had a role in glucose-induced insulin release by enhancing the depolarization of the voltage-gated Ca^2+^ channel, and offered protection against apolipoprotein CIII-induced β-cell apoptosis [58]. In contrast, the TTR monomer did not affect glucose-induced insulin release or apolipoprotein CIII-induced β-cell apoptosis [58]. TTR, transthyretin.

TTR also plays a role in glucose homeostasis in the liver and has potential beneficial effects in diabetes and its retinal complications (Figure 3) [63]. Diabetic retinopathy is a leading cause of blindness in the working-age population [137]. In a mouse model, TTR treatment significantly prevented the progression of retinal pathology [136]. The addition of TTR to cultures of human retinal microvascular endothelial cells mitigated the damaging effects of high glucose concentration *via* the VEGFA/PI3K/AKT pathway, suggesting this as a mechanism for the findings in mice [136]. In summary, evidence suggests an important role for tetrameric TTR in metabolic function and possible protection from diseases such as diabetes mellitus; this effect arises from its ability to contribute to glucose homeostasis and to protect against β-cell failure/destruction [58].

### Rheumatic and osteoarticular diseases

Rheumatic and osteoarticular diseases, which encompass inflammatory and degenerative conditions such as rheumatoid arthritis (RA), osteoarthritis, and osteoporosis [138], may be impacted by modulation of TTR. TTR may support the regulation of healthy bone mineralization; for example, research has shown that destabilized TTR, due to aging and other factors, distorts the morphology of calcium carbonate crystals *in vitro,* leading to amorphous and porous tissue [61]. Given the significance of calcium carbonate in maintaining bone constitution, these findings suggest the importance of TTR in bone formation and maintenance, and support the potential role of TTR in osteoporotic progression. A study that analysed multi-tissue TTR mRNA levels in 420,531 individuals from the UK Biobank revealed that rates of osteoporosis (and other poor health outcomes) were increased among individuals with raised hepatic *TTR* transcription, suggesting that ‘normal’ transcription of TTR is, in part, necessary for the maintenance of bone integrity [139]. TTR has been implicated as a biomarker for inflammation [140]. Relatedly, TTR expression has been linked to several rheumatic and osteoarticular diseases; for instance, in RA, TTR is considered an acute-phase response protein, as it is upregulated under inflammatory conditions, denoting a role for TTR as a biomarker in RA [139,141].

### Pregnancy

Regulation of systemic TTR and TTR expression by placental villous trophoblasts at the maternal-placental interface has been suggested as essential for the maintenance of healthy pregnancy and normal fetal development [50]. T_4_ is needed for fetal development and, in the first 14–16 weeks of gestation, the fetus is dependent on maternal T_4_ delivered by TTR synthesized in trophoblasts, after which time the fetus starts to produce its own T_4_ [142–145]. Disturbances to thyroid hormone transport and its metabolism are potentially associated with intrauterine growth restriction (IUGR) and miscarriage [142]. TTR expression in human placentas from mothers with babies affected by IUGR was found to be lower than that in control placentas [143]. The induction of IUGR in a rat model was associated with downregulation of TTR expression—mediated by an endogenous microRNA—in the labyrinth zone of the placenta, where gas and nutrient exchange occurs in relation to the placenta and trophoblasts, respectively [143]. Trophoblastic expression of TTR was also found to be significantly lower in placentas from women with miscarriages than in placentas from women undergoing elective pregnancy termination [142].

Systemic TTR is directly implicated in the multifactorial, hypertensive syndrome of preeclampsia [51,146], which affects 5–7% of pregnant women and is a leading cause of maternal death, severe maternal morbidity, and prematurity in the US [147]. The first stage of preeclampsia beginning early in the first trimester of pregnancy is described as abnormal placentation [147]. This is followed by uteroplacental ischaemia leading to the maternal syndrome in the late second and third trimesters, which is characterized by systemic vascular dysfunction, resulting in hypertension and proteinuria [147]. Using a mouse model, TTR was identified as a key amyloidogenic protein involved in the pathogenesis of preeclampsia [148]. Aggregates of TTR found in the trophoblasts of placentas from mothers with preeclampsia are believed to be a contributory cause of the pathophysiology [146]. TTR in serum from women with preeclampsia has shown a higher propensity to aggregate than TTR from women with healthy pregnancies [146,149]. Aberrant TTR from the serum of women with preeclampsia reproduced preeclampsia-like features in pregnant IL-10^–/–^ mice (a high-inflammation rodent model), and these were rescued by the administration of exogenous native human TTR [148]. Mice engineered to overexpress human TTR while their endogenous murine TTR was silenced developed a preeclampsia-like phenotype when pregnant, and deposits of TTR aggregates were found in the placenta [146]. One consequence of this could be a reduction in the ability of TTR to bind to and protect the vascular system from soluble endoglin, an anti-angiogenic factor positively associated with preeclampsia pathogenesis [148,150]. It has also been suggested that preeclampsia serum-mediated disruption in the communication between endothelial cells and trophoblasts, and the subsequent impairment of capillary tube formation, can be rescued by the addition of native TTR in an *in vitro* model [148]. Additional data *in vitro* and in mice have linked TTR aggregation with chronic hypoxia/reoxygenation stress; it is suggested that this stress produces a cellular environment conducive to tetramer dissociation and misfolding of the TTR monomers [146].

In one study, TTR expression was noted to be downregulated in syncytiotrophoblasts of placenta in women with preeclampsia compared with placentas from gestation-matched women with normal pregnancies [151]. The investigators speculated that any consequential reduction in T_4_ transport in the first trimester of pregnancy could impair the proliferation and differentiation of trophoblasts, adversely affecting placental development [151].

Another hypothesis that has emerged states that temporal modulation of the serum concentration of TTR during pregnancy is an adaptive response to protect against TTR misfolding and aggregation in an inflammatory placental microenvironment [51]. The serum concentration of TTR falls in the first 12–14 weeks of a normal human pregnancy but returns to prepregnancy levels by term [51]. Low serum TTR concentrations in IL-10^–/–^ mice were attributed to the absence of IL-10, an anti-inflammatory cytokine [51]. Overexpression of human TTR in transgenic mice led to fewer embryos and lower fetal weight, suggesting that downregulation of TTR is necessary for the maintenance of normal pregnancy and that inflammation may contribute to this downregulation, thereby mitigating the propensity for TTR to form cytotoxic aggregates in conditions of chronic hypoxia and inflammation [51].

In summary, it has been suggested placental TTR is essential for normal pregnancy and healthy fetal development [50]. Regulation of circulating TTR concentrations is especially important for pregnancy in the context of inflammatory cellular environments, which are conducive to harmful TTR aggregation [51]. Finally, TTR dysregulation leading to TTR aggregates in the placenta contributes to the pathogenesis of preeclampsia [146,148].

### Myogenesis

TTR plays a key role in the equilibrium between protein synthesis and degradation, serving as the primary measure of lean body mass resources [152]. Evidence from *in vitro* animal models suggests that TTR contributes to the development and regeneration of muscle cells, playing an important role in T_4_ delivery to the developing or injured tissue [59,60,64]. Indeed, knockdown of TTR in an *in vitro* animal cell model has been shown to impair muscle regeneration, indicating the role of TTR and thyroid hormone coordination in promoting muscle growth and development [59]. In animal and cell culture studies, TTR appears to participate in both the proliferation and differentiation of muscle cells, assisting in the delivery of T_4_ to maintain the required cellular pool of triiodothyronine for muscle development [59,60]. Furthermore, TTR facilitates the recruitment of myoblasts to areas of injury, in addition to cell-cycle progression and differentiation [59]. In humans, plasma TTR levels have proven to be an effective biomarker of sarcopenia [152].

### Mortality

Recent studies have underscored the significant negative association between TTR levels and mortality across several clinical populations. Lower TTR levels were linked with higher mortality in the general elderly population (potentially confounded by the correlation of TTR levels with nutritional status [153]), several oncological diseases, respiratory diseases, as well as cardiovascular diseases, and ATTR [9,52–56,154]. For example, Wang et al. reported that hospitalized elderly patients with the lowest TTR levels went on to have significantly higher all-cause mortality and hospital readmission compared with those with TTR concentration between the 5^th^ and 95^th^ percentiles [154]. A recent study that analysed genetic data from two prospective cohort studies of the Danish general population showed an association between TTR destabilization and all-cause mortality and cardiovascular mortality [49]. In addition, the prognostic value of TTR is also notable with respect to mortality in several forms of malignancy, for example in liver cancer and gastric cancer, in which low levels of TTR are associated with poor prognosis and increased mortality [55,56].

### Additional disease states

In several disease states, evidence concerning the physiologic role of TTR is too sparse or contradictory to allow for meaningful inferences concerning function. For example, TTR has been shown to interact directly with cancer cells and signalling pathways to regulate the tumour microenvironment, including the blood supply and the surrounding immune cells [155,156]. In lung cancer, there is mixed evidence as to whether TTR is beneficial or pathogenic. Some studies show that TTR suppresses lung cancer cells, arresting division, promoting apoptosis, and inhibiting growth in models of lung cancer [65,157]. However, other studies point to the involvement of TTR in intracellular processes that contribute to cancer progression. For example, it has been shown that TTR can stimulate the proliferation of Lewis lung carcinoma cells *in vitro* and increase the growth of lung tumours in a mouse model [155].

The role of TTR in renal and endocrine function also warrants further research. Altered expression of TTR could affect thyroid hormone levels, resulting in hypothyroidism or hyperthyroidism, and issues such as altered cardiac output and gastrointestinal dysfunction [158]. Maintaining thyroid hormone homeostasis involves a crucial interaction between TTR and megalin, an important endocytic receptor that binds TTR in a manner that is dependent on its tetrameric conformation [159]. Chronic kidney disease is often reported in patients with ATTR amyloidosis; however, the physiologic impact of TTR in relation to renal function and disease requires further characterization [160].

## Clinical considerations for treating ATTR amyloidosis

### Treatment landscape

Since the regulatory approval of the first ATTR-CM therapies in 2018, ATTR amyloidosis disease awareness, availability of targeted therapies, and the adoption of non-invasive diagnostic techniques have increased exponentially, resulting in patients being diagnosed earlier and living longer on therapies, with improved quality of life [18,161,162]. Several ATTR therapies are now approved and available, and numerous investigational treatments are in development [18]. These treatment approaches are, by their very design, intended to modulate TTR levels *via* TTR tetramer stabilization, TTR mRNA knockdown, *TTR* gene editing, or antibody-mediated amyloid removal [18].

#### TTR stabilizing therapies

TTR tetramer stabilizers are small molecule therapeutics that work by binding to TTR in its native tetrameric form and preventing the disassociation of the tetramer into pathogenic precursors of amyloid fibrils. Most recently, the US Food and Drug Administration (FDA), European Medicines Agency (EMA), UK Medicines and Healthcare products Regulatory Agency (MHRA), and Japanese Pharmaceuticals and Medical Devices Agency (PMDA) granted approval of acoramidis for the treatment of adults with ATTR-CM, both ATTRwt and ATTRv [163–166]. Acoramidis is a near-complete (≥ 90%), oral TTR stabilizer designed to mimic the stabilizing effects of the disease-protective gene variant p.T139M [167–170]. The FDA, EMA, MHRA, and PMDA have also approved tafamidis and/or tafamidis meglumine for ATTR-CM [171–174]. Tafamidis, a small molecule benzoxazole derivative, prevents the dissociation of wild-type TTR and variant TTR tetramers by binding the T_4_ binding site [172]. It was first approved by the EMA in 2011 for ATTRv with polyneuropathy, and subsequently approved by the FDA for ATTR-CM in 2019 [18,175]. As compared with tafamidis, acoramidis shows improved selectivity and increased binding potency for the TTR T_4_ binding site, mediated by key hydrogen bonds, enabling near-complete (≥ 90%) TTR stabilization independent of TTR genotype [170,176,177].

#### TTR knockdown therapies

A different therapeutic strategy for ATTR amyloidosis involves the knockdown of TTR by targeting the complementary mRNA within hepatocytes, promoting their degradation and thus stopping TTR at one of its sources [172,178]. This strategy aims to significantly reduce the production and circulation of TTR [172,179]. Small interfering RNAs and antisense oligonucleotides have been investigated for the treatment of ATTR *via* gene knockdown [172]. The small interfering RNAs patisiran and vutrisiran (delivered by intravenous infusion and subcutaneous injection, respectively), and the antisense oligonucleotides inotersen and eplontersen (delivered by subcutaneous injection), are FDA-approved therapies for ATTRv with polyneuropathy, with or without cardiac involvement [172,180–182]. Vutrisiran has also recently been approved by the FDA for the treatment of adults with ATTR-CM in both ATTRv and ATTRwt [181].

#### TTR gene editing via CRISPR-Cas9 therapies

Clustered regularly interspaced short palindromic repeats and associated Cas9 endonuclease (CRISPR-Cas9) is a gene editing approach, the aim of which is to permanently edit the *TTR* gene in hepatocytes, leading to a sustained decrease in the production of TTR [183]. NTLA-2001 (nexiguran ziclumeran or nex-z), uses lipid nanoparticles with liver tropism to release guide RNA and mRNA coding for Cas9 protein production, with the aim of achieving curative treatment for ATTR with a single administration [183,184].

#### ATTR amyloid depleting therapies

Another potential therapeutic strategy for ATTR focuses on clearing the deposition of disease-causing amyloid fibrils from affected tissues [18]. Two monoclonal antibodies currently under clinical investigation for depleting amyloid fibrils in humans are ALXN2220 (also known as NI006 and NI301A) and NNC6019-0001 [185]. ALXN2220 selectively binds with high affinity to an exposed epitope in misfolded TTR oligomers and aggregated TTR [186].

### Clinical considerations

A robust and varied compendium of evidence underscores the critical physiologic importance of TTR. Myriad organ systems, tissues, and cellular processes require TTR for homeostatic function. Further, the evidence suggests that TTR is broadly protective in numerous diseases [1,26]. In humans, destabilization of TTR can cause disease (e.g. ATTR), and stable TTR, in its native tetrameric form, can potentially mitigate disease (e.g. sequestration and proteolysis of Aβ) [11,17,46,49,97,98,100,104]. However, research is required to further elucidate the disease-modifying activities of TTR, particularly with respect to mitigating pathogenesis. With the recent emergence of targeted ATTR therapies that either preserve or diminish circulating levels of TTR and its activities, it is vital to assess long-term health consequences. The impact of TTR knockdown therapies on the prevalence or exacerbation of diseases in which TTR may have a protective role should continue to be interrogated in real-world datasets. However, despite the uncertainties surrounding prolonged TTR knockdown, available treatments significantly improve disease outcomes in patients with ATTRv polyneuropathy or ATTR-CM compared with placebo [181,182,187]. Overall, the ubiquitous function of TTR in human biology should be considered as part of comprehensive clinical decision-making when selecting therapies for patients with ATTR amyloidosis. Here, we discuss potential clinical considerations of TTR-directed therapies for ATTR in the context of the summarized literature.

#### Neurodegenerative disease and cognitive function

The neuroprotective role of TTR is well established, with evidence of beneficial physiologic activity in AD, Lewy body disorders (such as Parkinson’s disease), and TDP-43–associated neuropathologies [44,45,47,96]. Further, evidence suggests that TTR may promote neuritogenesis, angiogenesis, and cognitive function, specifically age-related cognitive performance [35]. However, additional evidence is required to demonstrate the clinical benefits in human cohorts, including those with TTR-associated diseases such as AD and the general population. It is clearly important to understand whether long-term TTR knockdown or genetic deletion may negatively impact cognitive function. To this end, we suggest that systematic cognitive assessments among patients receiving TTR stabilizers or knockdown therapies will provide useful data and allow clinicians to make an informed clinical decision regarding ATTR therapies for their patients. While the long-term cognitive effects of TTR knockdown remain uncertain, there is currently no conclusive evidence demonstrating harm in patients with ATTRv polyneuropathy receiving TTR knockdown therapy over the last 10 years. The number of patients who have received long-term treatment, however, is small [188]. Another interesting avenue of investigation is related to BBB dysfunction and the subsequent increase in vascular permeability seen in AD [85]; would this increased permeability extend to the ability of ATTR therapies to cross the BBB, and if so, how this would alter the clinical course of AD and other neurodegenerative disorders. Relatedly, leptomeningeal ATTR is likely to increase in incidence with the currently improved ATTR treatments directed at the liver [189]. Patients with ATTRv generally have lower serum TTR levels than non-ATTRv controls [9,190], and CNS involvement, including cognitive decline, is a common complication of ATTRv [191]. The current treatments available for ATTR have no or poor CNS penetration [192,193]. One potential treatment that could be developed is intrathecal TTR silencing [194], but assessing the effects of TTR knockdown on AD development in this setting would be critical.

#### Cerebrovascular and cardiovascular disease

Findings from several population cohort studies link low TTR levels with increasing risks of heart failure, coronary artery disease, all-cause mortality, and cardiovascular mortality [9,48,132]. However, it is important to consider that a proportion of the data may be directly attributable to ATTR-CM and not the result of a distinct vascular disorder. Recent observations in the UK Biobank showed that carriers of the *TTR* pathogenic variant p.V142I have reduced TTR levels, with low TTR levels associated with increased risks of heart failure, cardiovascular mortality, and all-cause mortality [9]. Using data from Danish cohort studies, it was determined that patients who are heterozygous for the stabilizing p.T139M *TTR* variant have increased TTR levels and decreased risks of cerebrovascular disease and all-cause death [49,54]; however, these data were not reproduced in the UK Biobank, in which p.T139M was not associated with protection against cerebrovascular disease or death [134]. Nevertheless, it is pertinent to determine the extent to which findings in the Danish cohort studies are generalizable to the global population; additional population-level data could inform whether data obtained from the Danish cohorts are region-specific phenomena.

#### Metabolism and metabolic disease

Evidence shows that TTR supports glucose homeostasis, both in the liver and in pancreatic β-cells; however, much of this evidence is from *in vitro* studies and has not been shown clinically [58,63]. It is currently unknown whether patients with ATTR have abnormal glucose homeostasis driven by a deficiency in TTR and are, therefore, at a higher risk of developing diabetes and other metabolic diseases. As such, there would be insufficient evidence to modify the approach to ATTR treatment on the basis of metabolic disease status. Future research could investigate the potential risk and exacerbation of metabolic diseases in ATTR patients in a real-world setting, as well as the long-term impact on patients with ATTR. Long-term data on how TTR therapies impact patients with diabetes or other cardiometabolic disorders are required to inform clinical decision-making.

#### Rheumatic and osteoarticular diseases

Given the important role of TTR in bone mineralization and the fact that frailty in patients with ATTR may predispose them to falls and fractures, the long-term impact of modulating TTR levels *via* therapy should be investigated [61,195]. Bone fractures subsequent to osteoporosis could significantly impair quality of life and increase the risk of death [196]. Evidence suggests that stable TTR is necessary for healthy bone tissue development and that there is a relationship between TTR levels and osteoporosis; therefore, it is essential to investigate whether long-term suppression of TTR levels has an impact on bone health [139,197]. This will elucidate if patients receiving TTR-directed therapies require long-term bisphosphonate therapy to preserve bone integrity. The potential adverse role of TTR in RA also warrants further investigation.

#### Pregnancy

TTR dysregulation has been shown to contribute to the pathogenesis of preeclampsia [146,148]. Despite this evidence, it remains to be determined whether women harbouring pathogenic *TTR* variants have higher rates of preeclampsia or IUGR than those without pathogenic variants. More broadly, studies are needed to establish whether *TTR* genotype (including stabilizing and pathogenic variants) influences complication rates in pregnancy and whether gestational history influences ATTR outcomes later in life. Preeclampsia is known to be a risk factor for long-term cardiovascular disease [198]. The question then arises of whether TTR dysregulation that commences during pregnancy in women with preeclampsia persists after delivery and, if so, for how long.

Studies are required to determine the effect of TTR-modulating therapies on pregnancy. Animal models of therapeutic TTR knockdown in pregnancy should be conducted to assess its effects on uterine and placental expression of TTR. Observational studies in women who have received TTR therapies prior to conception could inform the washout period for these drugs before a planned pregnancy and the potential for longer-term sequelae of prepregnancy TTR silencing.

#### Myogenesis

The evidence we reviewed points to a vital function of TTR in muscle development, regeneration, and response to injury [59,60,64]. This raises an important clinical question of whether chronic suppression of TTR, resulting from TTR knockdown therapies or untreated ATTR, would be deleterious for muscle tissue and function. Muscle weakness, atrophy, and wasting are common in ATTR, and it is possible that this could be exacerbated by chronic low TTR levels. Though it may be premature to modify ATTR treatment on the basis of these data, close clinical monitoring of muscular health and function may enable earlier detection of deleterious effects. Clinical research assessing whether ATTR therapies impact skeletal muscle health and repair would help inform future ATTR treatment decisions.

## Discussion

As we look to the future, understanding the role of TTR in normal physiology and pathology is essential for improving clinical care for patients with ATTR and beyond. Further elucidating the neuroprotective function of TTR and its role in the regeneration of bone, muscle, and neural tissue could inform on personalized strategies in the treatment of ATTR and possibly lead to novel therapeutic interventions in other diseases. ATTR amyloidosis is being diagnosed earlier and with greater frequency, and the therapeutic landscape is broadening with a multitude of treatment modalities. Serum TTR is significantly decreased in ATTRv due to the presence of destabilizing variants and is at the lower end of normal in ATTRwt, compared with ‘healthy’ individuals [53,199,200]. In ATTRv, low levels of serum TTR are present owing to pathogenic variants that lead to greater tetramer dissociation than wild-type, and are associated with earlier disease onset and more rapid progression of the disease [48,201,202]. TTR knockdown, however, leads to clinical improvements in ATTRv polyneuropathy and ATTR-CM [22,181,182,187]. Moreover, treatment-related increases in serum TTR observed with TTR stabilizers are associated with lower all-cause death and cardiovascular-related hospitalization and mortality in patients with ATTR-CM [203,204]. Therefore, sTTR is emerging as a useful biomarker for TTR stability, whether intrinsic or pharmacologically modified, in response to therapy [133]. Thus, routine evaluation of sTTR levels may serve as a potential and informative biomarker of drug efficacy and treatment response at an individual level, reflecting TTR stability and a decrease in new amyloid formation in patients with amyloidosis. Patients undergoing treatment for ATTR have increased longevity, which equates to more time on therapy. Current interventions aimed at silencing TTR mRNA are meant to be indefinite. It remains unknown, given the important physiologic role of TTR, how decades of suppression might influence physiologic functions. Knowing that 3% to 4% of African Americans carry the p.V142I mutation that may lead to ATTR-CM [205,206], there are likely over 1,000,000 individuals in the United States alone who could end up utilizing long-term TTR suppression therapy given its proven benefits in prolonging survival [182]. Long-term safety monitoring to detect early declines in bone health, cognitive dysfunction, or changes in blood glucose are not currently part of FDA labelling for these agents but carry a significant theoretical risk given the known and discussed homeostatic role of TTR in associated tissues and should be monitored as part of future clinical trials, especially those testing ATTR therapies in younger or even presymptomatic individuals. Overall, contemporary treatment choices for ATTR should reflect what is known of TTR function and be individualized for specific patient contexts. Additional research, assessing the potential long-term impact of chronic TTR suppression from knockdown therapies may empower providers to critically select a therapy, with either a TTR stabilizer or silencer depending on each individual patient’s substrate. Critical questions, such as whether chronically low TTR levels predispose patients with ATTR to other serious and fatal medical conditions, must be explored. It is imperative to investigate the relationship between the duration and degree of TTR suppression and the associated risks of serious disease.

## Conclusions

This comprehensive review offers a synthesis of evidence highlighting the diverse and critical roles of TTR across numerous physiologic functions and various disease conditions (see Supplementary Table). TTR is essential for a broad spectrum of physiologic processes, affecting neurodegenerative disorders, cognitive health, metabolic balance, pregnancy, and bone, muscle, vascular, and neural tissue regeneration. Substantial progress has been made in elucidating the important and diverse activities of TTR; however, further investigation is crucial to fully comprehend the complex mechanisms by which therapies for ATTR impact patient health. Now that we have entered an era in which long-term TTR suppression in patients with ATTR is available, continued research into the clinical consequences of lowering TTR becomes vital. The rapidly expanding landscape of TTR-targeted therapies suggests a possible future role for personalized medicine, taking into consideration the mechanism of action alongside patient comorbidities. However, before this becomes a reality, much needs to be learned about the role of TTR in disease states and whether *in vitro* and animal model data are replicated further in human physiology. It is imperative to continue elucidating the role of TTR in disease pathophysiology prior to assigning individual therapeutic strategies. Research into potential long-term toxicities of chronic TTR knockdown, along with the benefits in ATTR amyloidosis, is increasingly important. We must delve deeper into the physiologic functions of TTR and the consequences of prolonged alteration of TTR levels on patient outcomes.

## Supplementary Material

Supplemental Material

Supplemental Material

## Data Availability

Data sharing is not applicable to this article as no new data were created or analysed in this study.
