# Supplementary figures and images for: Unravelling the myriad physiologic roles of transthyretin: critical considerations for treating transthyretin amyloidosis
Source: Ann Med. 2025 Jul 27;57(1):2536755. doi: 10.1080/07853890.2025.2536755 (PMC12305880; doi:10.1080/07853890.2025.2536755)

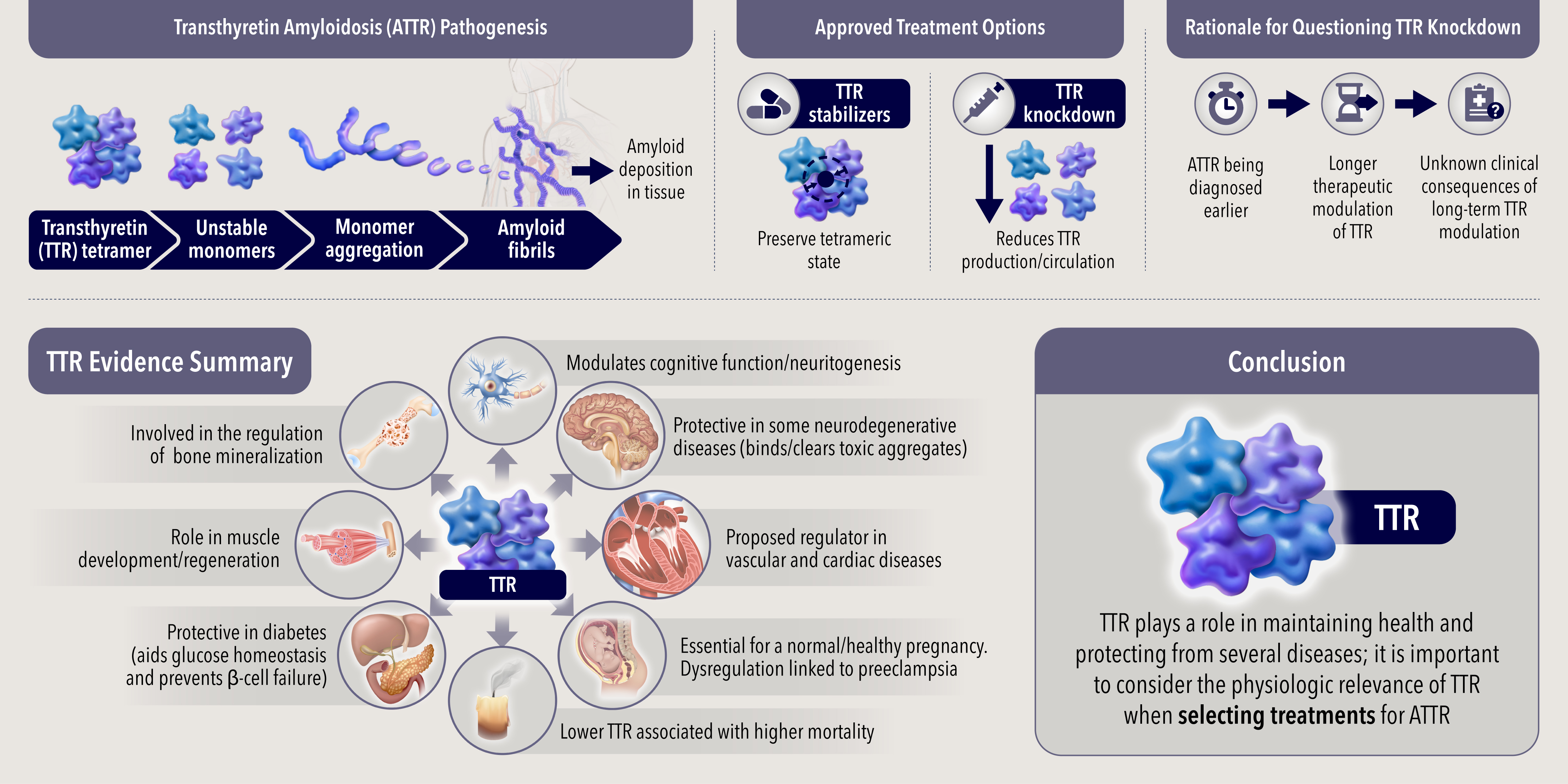

Supplement: Supplemental Material [file IANN_A_2536755_SM2749.jpg]
