# Supplementary material for: Unravelling the myriad physiologic roles of transthyretin: critical considerations for treating transthyretin amyloidosis
Source: Ann Med. 2025 Jul 27;57(1):2536755. doi: 10.1080/07853890.2025.2536755 (PMC12305880; doi:10.1080/07853890.2025.2536755)
Supplement: Supplemental Material [file IANN_A_2536755_SM1838.docx]

# Appendix

**Supplementary Table.** List of articles describing TTRs role in heath and disease

**Supplementary Table.** List of articles describing TTRs role in heath and disease

| **Area** | **Evidence** |
| --- | --- |
| Alzheimer’s disease | Alemi M, et al. Sci Rep. 2016;6:20164^a^  Alemi M, et al. CNS Neurosci Ther. 2017;23(7):605–619^a–c^  Buxbaum JN. J Integr Neurosci*.* 2023;22(6):158. doi: 10.31083/j.jin2206158.^d^  Cheng Y, et al.Transl Neurodegener. 2020;9(1):16^d^  Corino C, et al. Mol Neurobiol. 2025;62(3):2945–2954^d^  Costa R, et al. PLoS One. 2008;3(8):e2899^e^  Du J, et al. Protein Eng Des Sel. 2012;25(7):337–345^e,f^  Ghadami S.A, et al. Biomacromolecules.  2020;21(3):1112–1125^e,f^  Gião T, et al. Alzheimers Res Ther. 2021;13(1):143^b,c^  Gloeckner SF, et al. J Alzheimers Dis. 2008;14(1):17–25^e,g^  Kanekiyo T, Bu G. Front Aging Neurosci. 2014;6:93^d^  Li X, Buxbaum J. Mol Neurodegener. 2011;6:79^d^  Li X, et al. J Neurosci. 2013;33(50):19423–33^a,f^  Mangrolia P, et al. Protein Eng Des Sel.  2016;29(6):209–218^e,f^  Nakamura M, et al. Arch Gerontol Geriatr. 2023;115:105226^β^  Pedrero-Prieti CM, et al. Clin Proteomics*.* 2020;17:doi: 10.1186/s12014-12020-09276-12019^d^  Perrin RJ, et al. PLoS One*.* 2011;6(1):e16032^e,g^  Petralla S, et al. Pharmaceutics. 2024;16(7):948^d^  Rejc L, et al. J Alzheimers Dis. 2020;77(1):99–112^a–c,e^  Ribeiro CA, et al. Curr Alzheimer Res. 2012;9(8):881–889^e,g^  Ribeiro CA, et al. PLoS One. 2012;7(9):e45368^a,e^  Ribeiro CA, et al. J Alzheimers Dis. 2014;39(2):357–370^b,c^  Rios X, et al. Sci Rep. 2019;9(1):13672^b,c^  Schultz K, et al Eur J Neurol*.* 2010;17(3):456–460^g^  Schwarzman AL, et al. Proc Natl Acad Sci U S A. 1994;91(18):8368–8372^b,e^  Serot JM, et al. J Neurol Neurosurg Psychiatry. 1997;63(4):506–508^g^  Silva CS, et al. Neurobiol Aging. 2017;59:10–14^a,e^  Velayudhan L, et al. J Alzheimers Dis. 2012;28(2):369–375^e,g^  Zareba N, Kepinska M. Int J Mol Sci. 2020;21(23):9003^d^ |
| Lewy body disorders | Sárkány Z, et al. bioRxiv. 2023;08.10.552896.  doi: 10.1101/2023.08.10.552896^a,e^ |
| TDP-43–related neuropathologies | Chu Y-P, et al. Brain. 2023;146(5):2089–2106^a–c,e^  Chu Y-P, et al. Autophagy. 2023;19(8):2403–2405^d^ |
| Cognitive function and neuritogenesis | Araghi M, et al. Neurol Sci. 2021;42(12):5093–5100^e,g^  Brouillette J, Quirion R. Neurobiol Aging.  2008;29(11):1721–1732^a–c,e^  Buxbaum JN, et al. Neuroscience. 2014;275:352–64^b,c^  Fleming CE, et al. J Neurochem, 2007;103(2):831–839^a–c^  Fleming CE, et al. J Neurosci. 2009;29(10):3220–3232^a–c^  Fleming CE, et al. Prog Neurobiol. 2009;89(3):266–276^d^  Gloeckner SF, et al. J Alzheimers Dis. 2008;14(1):17–25^e,g^  Gomes JR, et al. Cell Death Differ. 2016;23(11):1749–1764^a,c^  Leirós M, et al. Front Aging Neurosci. 2022;14:880405^g^  Mar FM, et al. Future Neurol. 2009;4(6):723–730^d^  Nakamura M, et al. Arch Gerontol Geriatr. 2023;115:105226^g^  Ribeiro CA, et al. Curr Alzheimer Res. 2012;9(8):881–9^g^  Sousa JC, et al. Neurobiol Learn Mem. 2007;88(3):381–5^b,c,e^  Tien YT, et al. Sci Rep. 2019;9(1):18691^g^  Velayudhan L, et al. J Alzheimers Dis. 2012;28(2):369–75^e,g^  Xiong Y, et al. Curr Opin Investig Drugs. 2011;11:298–308^d^ |
| Cerebrovascular and cardiovascular disease | Christoffersen M, et al. JAMA Cardiol. 2024; e244102.  doi: 10.1001/jamacardio.2024.4102^e,g^  Gomes JR, et al. Cell Death Differ. 2016;23(11):1749–1764^a,c^  Greve AM, et al. JAMA Cardiol. 2021;6(3):258–266^e,g^  Hornstrup LS, et al. Arterioscler Thromb Vasc Biol. 2013;33(6):1441–1447^e,g^  Monu, et al. Mediators Inflamm. 2020:3429541^e,g^  Parker MM, et al. Amyloid. 2020;27(3):184–190^e,g^  Shetty NS, et al. Nat Commun. 2024;15(1):6221^e,g^ |
| Metabolism and metabolic disease | Alemi M, et al. Int J Mol Sci. 2021;22(11):6073^a–c^  Liu L, et al. Invest Ophthalmol Vis Sci. 2024;65(1):45^a–c^  Hendrick AM et al. Prim Care. 2015;42(3):451–64^d^  Refai E, et al. Proc Natl Acad Sci U S A. 2005;102(47):17020–17025^a,c,g^ |
| Rheumatic and osteoarticular diseases | Monu, et al. J Inflamm Res. 2021;14:5581–5596^a,e,g^  Pathak GA, et al. Amyloid. 2022;29(2):110–119^e,g^  Wieczorek E, et al. Biochim Biophys Acta Gen Subj. 2019;1863(2):313–324^a^ |
| Pregnancy | Calvo RM, et al. J Clin Endocrinol Metab. 2002;87(4):1798–1777^g^  Cheng S, et al. Hypertension*.* 2022;79(8):1738–1754^a–c^  Cheng S, et al. Biology (Basel)*.* 2023;12(8):1048.  doi: 10.3390/biology12081048^b,c,g^  Kalkunte SS, et al. Am J Pathol*.* 2013;183(5):1425–1436^a,c^  Medegan Fagla B, Buhimschi IA. Molecules*.* 2024;29(3):610. doi: 10.3390/molecules29030610^d^  Obregon MJ, Endocr Dev. 2007:10:86–98^d^  Rana S, et al. 2019;124(7):1094–1112^d^  Saha S, et al. Sci Rep*.* 2017;7(1):16548.  doi: 10.1038/s41598-017-16566-0^a,c^  Young M, et al. Mol Cell Endocrinol*.* 2023;562:111851. doi: 10.1016/j.mce.2023.111851^a,g^  Yu Z, et al. Reprod Biol Endocrinol*.* 2023;21(1):108.  doi: 10.1186/s12958-023-01142-1^b^  Zhu L, et al. Taiwan J Obstet Gynecol*.* 2016;55(3): 385–389^a,e,g^ |
| Myogenesis | Ingenbleek Y. Nutrients. 2019;11(4)^d^  Lee EJ, et al. Int J Mol Sci. 2017;18(1):115^a^  Monk JA, et al. Am J Physiol Endocrinol Metab. 2013;304(1):E23–31^a–c^  Pokharel S, et al. In Vitro Cell Dev Biol Anim. 2014;50(8):756–765^a^ |
| Mortality | Christoffersen M, et al. JAMA Cardiol. 2024: e244102. doi: 10.1001/jamacardio.2024.4102. Epub ahead of print ^e,g^  Fujita K, et al. Respir Investig. 2024;62(5):889–96^g^  Hanson JLS, et al. Circ Heart Fail. 2019;11(2)^g^  Hornstrup LS, et al. Arterioscler Thromb Vasc Biol. 2013;33(6):1441–7^e,g^  Ingenbleek Y. Front Med. 2022;16(4):540–50^d^  Qiao W, et al. 2020;72(6):909–16^d^  Shetty NS, et al. Nat Commun. 2024;15(1):6221^e,g^  Shimura T, et al. J Surg Res. 2018;227:145–50^g^  Wang T, et al. BMC Geriatr. 2024;24:893^g^ |

TDP-43, trans-activation response DNA-binding protein 43; TTR, transthyretin.
^a^*In vitro* data; ^b^*In vivo* data; ^c^mouse/rat models; ^d^review article; ^e^proteomics; ^f^mutagenesis; ^g^human data.
